# Neurological emergency at the COVID-19 pandemic: report from a referral hospital in Eastern Piedmont, Italy

**DOI:** 10.1007/s10072-022-05895-2

**Published:** 2022-01-18

**Authors:** Claudia Varrasi, Thomas Fleetwood, Fabiola De Marchi, Domizia Vecchio, Eleonora Virgilio, Luigi Mario Castello, Gian Carlo Avanzi, Pier Paolo Sainaghi, Letizia Mazzini, Roberto Cantello

**Affiliations:** 1 Department of Neurology and ALS Centre, Translational Medicine, University of Piemonte Orientale, Maggiore Della Carità Hospital, Corso Mazzini 18, 28100 Novara, Italy; 2 Emergency Department, Translational Medicine, University of Piemonte Orientale, Maggiore Della Carità Hospital, Novara, Italy; 3 Internal Medicine, A.O. Santi Antonio E Biagio E Cesare Arrigo, Alessandria, Italy

**Keywords:** Pandemic, COVID-19, Stroke Unit, Acute neurology, Management

## Abstract

**Background:**

The pandemic implied dramatic changes in public health assets. In Italy, some Stroke Units were transformed into sub-intensive COVID-19 Units, making the management of neurological patients demanding. We described how the flow of neurological emergencies was affected by the pandemic impact.

**Methods:**

We analyzed accesses to the Emergency Department (ED) of the “Maggiore della Carità” Hospital, Piedmont, Italy, during a period of 8 months (COVID time; March to May 2020 and October 2020 to February 2021) and analyzed the admissions to the Neurology Unit and the underlying diagnosis. We also evaluated potential changes in the treatment of acute ischemic stroke in the same period. These variables were compared with two equivalent periods of time (2019–2020; 2018–2019).

**Results:**

During the COVID time, there was a clear-cut reduction of the total ED accesses compared to NoCOVID times. However, admissions for acute neurological conditions showed a mild but non-significant decrease (6.3%vs.7.3%). The same applied to acute ischemic stroke, which represented the most common condition (47.7%). The proportion of patients who underwent emergent reperfusion therapies remained unchanged. Furthermore, no difference was found in door-to-needle and door-to-groin intervals between COVID time and NoCOVID times. On the contrary, the onset-to-door interval was significantly longer during the COVID time (*p* value: 0.001).

**Discussion:**

While the percentage of admissions following an ED access grew dramatically, those to the Neurology Unit showed overall only a slight non-significant decrease. This finding implicitly reflects the serious and urgent nature of many neurological diseases, compelling people to access EDs at any time.

## Introduction

The coronavirus disease-19 (COVID-19) is the acute respiratory syndrome responsible for the severe pandemic declared at the beginning of March 2020 (https://www.who.int/director-general/speeches/detail/who-director-general-s-opening-remarks-at-the-media-briefing-on-covid-19---11-march-2020, checked on June 22, 2021). As universally recognized, the pandemic implied dramatic changes in public health assets, mainly due to a massive hospitalization and intensive care of COVID-19 patients. Outpatient and routine activities were drastically reduced or abolished. Revolutionary changes affected many wards and units, which were re-converted to host COVID-19 patients. This was particularly true in the Piedmont region, North-Western Italy, one of the areas most affected by the pandemic. Here, several hub-and-spoke models of care were compromised, among which the stroke network. Established guidelines for stroke treatment were challenged. Some Stroke Units were transformed into sub-intensive COVID-19 Units, as was the case for the tertiary referral “Maggiore della Carità” Hospital, Novara, Piedmont, Italy. This hospital serves a roughly 365,000-inhabitant community as a hub for intravenous thrombolysis and about 870,000 inhabitants for mechanical thrombectomy. Nevertheless, acute stroke patients were allocated in care areas which not always reached the expected standards.

Prompted by these dramatic changes, we wanted to describe how the flow of overall neurological emergencies was affected by the impact of the COVID-19 pandemic, with an emphasis on the ischemic stroke.

To this aim, we analyzed the accesses to the Emergency Department (ED) of the “Maggiore della Carità” Hospital, Novara, Italy, and analyzed, among other variables, the admissions to the Neurology Unit and the underlying diagnosis. We also evaluated potential changes in the treatment of acute ischemic stroke. Appropriately defined time windows belonging to previous (NoCOVID) years served as controls.

## Methods

### Setting of care

We recorded and described the main changes which the Neurology Unit and its personnel underwent during the considered pandemic epochs.

### Survey on neurological emergencies

We conducted a retrospective study at the “Maggiore della Carità” Hospital, Novara, Italy. At first, we analyzed an index period from October 1, 2019, to February 28, 2021. Such period included a “pre-COVID” interval (ending up on February 29, 2020), which served as a baseline for the subsequent definition of the COVID-19 pandemic peaks.

**Fig. 1:**
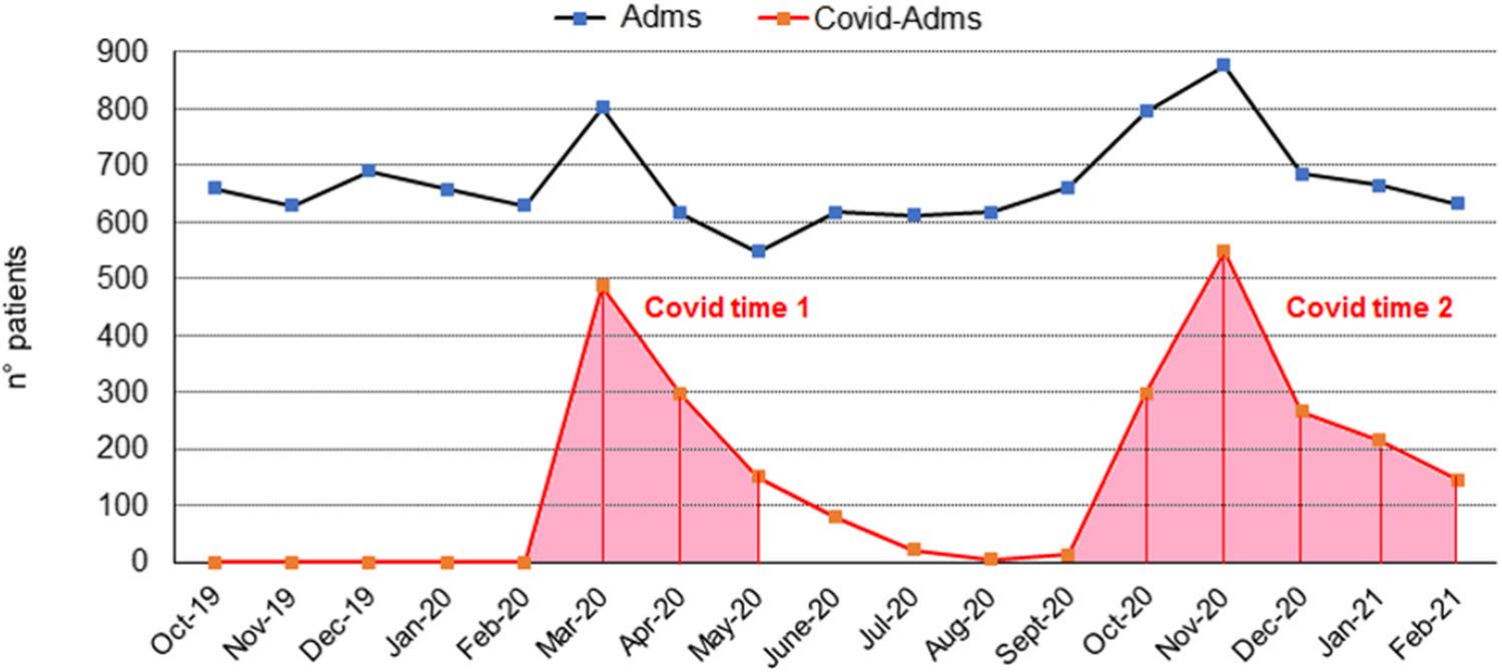
Time behavior of the overall hospital admissions (Adms) and admissions to the COVID wards (COVID-Adms) after an access to the Emergency Department at our Institution. The timespan goes from October 2019 to February 2021, included. Two phases with a consistent amount of COVID-Adms are seen, COVID time—1 and COVID time—2

During the index period, we analyzed all accesses to the adult ED through the computer program “PSNet,” operating on the local intranet. At first, we counted the total number of hospital admissions (Adms) per month, and the number of admissions to COVID wards (COVID-Adms) per month. This generated a graph highlighting the rising, peak, and decay of the COVID-Adm number, thereby defining two “COVID times” (Fig. 1). The first one went from March to May 2020 (both included; COVID time 1 = 3 months). Its peak occurred in March 2020. The second one went from October 2020 to February 2021 (both included; COVID time 2 = 5 months). Its peak occurred in November 2020. Termination of COVID times was forcefully defined as the end of February 2021, when the observational study was stopped. At that moment, COVID-Adms were 150/month.

By summing up COVID times 1 and 2, we then obtained an overall “COVID time,” lasting 8 months. “NoCOVID time—1” and “NoCOVID time—2” included the same 8 months, as extracted from the years 2019–2020 and 2018–2019.

Analyzing the COVID time, we measured how many ED accesses and total admissions to the hospital wards (Adms) had occurred therein. Also, we investigated the admissions to the Neurology Unit (Neu-Adms) and their demography. Subsequently, these variables were compared with NoCOVID time—1 and NoCOVID time—2.

Considering Neu-Adms, we further analyzed the clinical records to identify the final diagnosis for each case, based on the International Classification of Diseases, 9th revision, Clinical Modification (ICD-9-CM) (Table 1) [1].

**Table 1:** Classification of neurological disorders considered in the study, based on International Classification of Diseases, 9th revision, Clinical Modification (ICD-9-CM) [1]

	Diagnosis
Cerebrovascular diseases**(ICD: 431, 433, 434, 435)**	Ischemic stroke, minor stroke, and transient ischemic attacks (TIAs); hemorrhagic stroke
Epileptic diseases**(ICD: 345)**	Epileptic disorders, status epilepticus
Tumors**(ICD: 191, 192.1, 198.3, 225.2)**	Cerebral tumors (including metastases)
Peripheral nervous system diseases**(ICD: 356, 357, 358, 359, 386)**	Neuropathy, myasthenia, myopathy, and vertigo
Miscellaneous**(ICD: 346, 332, 340, 290, 293.1, 331, 335.2,)**	Headache, movements disorders, multiple sclerosis, delirium, and psychogenic disease, amyotrophic lateral sclerosis, apart from rare neurological disease

Furthermore, we then investigated if treatment of acute ischemic stroke had undergone any changes during the COVID time. We thus recorded the number and type of reperfusion therapies carried out in the whole patient group, compared with the NoCOVID times. We finally analyzed the stroke cases which were admitted directly to our hub center. Among these, we searched for differences in the onset-to-door (OTD), door-to-needle (DTN), and door-to-groin (DTG) times.

### Ethical considerations.

The study was entirely retrospective. The protocol was approved by the Institutional Review Board (Comitato Etico Interaziendale Novara; IRB code CE 97/20) and conducted in strict accordance with the principles of the Declaration of Helsinki. Data was anonymized. Due to the retrospective design, no risks were expected for the patients, and the results of the study did not impact their diagnosis, prognosis, or management.

### Data availability

Datasets generated during this study are available from the corresponding author on reasonable request.

### Statistics

Categorical variables were reported as numbers and percentages, and were compared by chi-squared analyses. Continuous variables were reported as mean ± standard deviation, and compared through analysis of variance (ANOVA). The threshold for statistical significance was set at *p* value < 0.05, with a Bonferroni correction for multiple comparisons. All statistical procedures were carried out with SPSS Version 25.0 (SPSS Inc., Chicago, IL, USA).

## Results

### Setting of care

During the pandemic epochs considered, the Neurology Unit was transformed in a medium-to-low-intensity COVID Unit. The Stroke Unit was transformed into a sub-intensive COVID Unit. Acute stroke patients were initially hosted in a sub-intensive neurosurgical area, and then in an ordinary neurosurgical ward. Here, extra facilities for monitoring and nurse assistance were provided, though the paramedics were not specifically trained. Non-stroke patients were initially mixed to neurosurgical patients. Then, they were transferred, often in small groups, to other wards (e.g., gastroenterology, orthopedics, otolaryngology). Neurologists were engaged in cyclical duties in COVID wards 24/7, but no one was full-time bound to COVID Units.

### Neurological emergencies

During the “COVID time,” there was a clear-cut reduction of the total ED accesses, in comparison with both NoCOVID time—1 and NoCOVID time—2 (reduction of 45% compared to NoCOVID times). By contrast, the total number of Adms showed a slight non-significant increase. Thence, the proportion of patients who were admitted after an ED access was strongly enhanced (chi: 1225; *p* value < 0.001), obviously due to the large number of COVID-Adms (Fig. 1; Table 2). Besides, the number of Neu-Adms remained virtually unchanged, and so did their demographic characteristics (Table 2).

**Table 2:** Accesses to the Emergency Department (ED) and overall hospital admissions (Adms). Admissions to the Neurology Unit (Neu-Adms) and to COVID wards (COVID-Adms)

	ED accesses	Adms	NEU-Adms	COVID-Adms
	(*n*)	(*n*, % ED acc.)	(*n*, % Adms; M/F; age ± SD)	(*n*, % Adms)
**COVID** time	17.942*	5.626; 31.3%*	354; 6.3% (164/190; 72.2 ± 15.7)	2.405; 42.7%
**NoCOVID** time—1	31.478	5.329; 16.9%	383; 7.2% (183/200; 71.8 ± 15.5)	
**NoCOVID** time—2	32.241	5.194; 16.1%	386; 7.4% (190/194; 71.8 ± 15.0)	

*p value < 0.001, COVID vs NoCOVID times

**Fig. 2:**
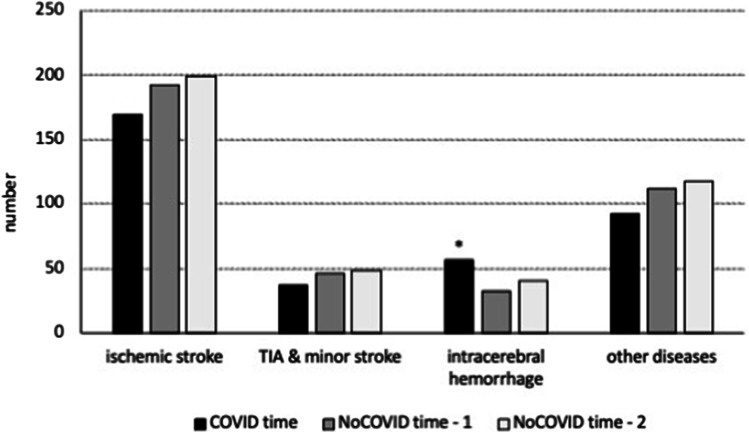
Number of admitted patients to the Neurology Unit in the COVID time, compared to NoCOVID time—1 and NoCOVID time—2. In this figure, we classified neurological conditions in ischemic stroke, transient ischemic attack (TIA) and minor stroke, intracerebral hemorrhage, and other diseases (epileptic diseases, tumors, peripheral nervous system diseases, miscellaneous). *p value: 0.02, COVID vs NoCOVID times

During COVID time, most Neu-Adms were represented by acute ischemic stroke cases, and, in spite of a slight reduction, this occurrence bore no significant difference from NoCOVID times (Table 3; Fig. 2). In addition, a separate analysis of COVID time 1 reported 57 ischemic stroke cases, out of 127 Neu-Adms (44.8%). The corresponding value for COVID time 2 was 49.4%, with no statistically significant difference (chi: 0.15; *p* value: 0.90). Even a comparison of the sole COVID time 1 vs the NoCOVID times led to negative results (chi: 1.44; *p* value: 0.50).

**Table 3:** Admissions to the Neurology Unit (Neu-Adms) comparing the COVID time and NoCOVID times. *TIA*, transient ischemic attack; *PNS*, peripheral nervous system; *Miscell*., miscellaneous

Neu-Adms	Ischemic stroke	TIA and minor stroke	Intracerebral hemorrhage	Epileptic disease	Tumors	PNS diseases	Miscell
(*n*)	(*n*, % Neu-Adms)						
**COVID** time (354)	169; 47.7%	37; 10.5%	56; 15.8%*	45; 12.7%	15; 4.2%	9; 2.5%	23; 6.5%
**NoCOVID** time—1 (383)	192; 50.1%	46; 12%	33; 8.6%	39; 10.2%	10; 2.6%	18; 4.7%	45; 11.7%
**NoCOVID** time—2 (386)	199; 51.6%	48; 12.4%	41; 10.6%	43; 11.1%	12; 3.1%	23; 6%	20; 5.2%

*p value: 0.02, COVID vs NoCOVID times

Interestingly, among the final diagnoses, we detected a significant increase in hemorrhagic stroke (chi: 7.69; *p* value: 0.02; Table 3, Fig. 2).

Finally, there was a trend to a reduction of the group termed “peripheral nervous system disease” (chi: 4.73; *p* value: 0.09). Here, the diagnosis “vertigo” was no longer represented (Table 3).

### Treatment of ischemic stroke

Among all ischemic strokes, the percentage of patients who underwent emergent reperfusion therapies remained unchanged during the COVID time (Table 4). Even, a slight non-significant increase was seen in patients treated by primary mechanical thrombectomy (MT) (chi: 4.30; *p* value: 0.10).

**Table 4:** Acute treatment of ischemic stroke. Number and percentage of reperfusion therapies subdivided into intravenous, mechanical, and “bridging therapy”

Ischemic strokes (all)	Emergent reperfusion therapies (all)	Intravenous thrombolysis (IVT) alone	Mechanical thrombectomy (MT) alone	Bridge therapy (BT)
(*n*)	(*n*, % ischemic strokes)			
**COVID** time (169)	68; 40.2%	21; 12.4%	28; 16.6%	19; 11.2%
**NoCOVID** time—1 (192)	66; 34.4%	23; 12%	26; 13.5%	17; 8.9%
**NoCOVID** time—2 (199)	65; 32.7%	27; 13.6%	17; 8.6%	21; 10.6%

We thus selected a subgroup of patients primarily referred to our hub center, to measure the onset-to-door (OTD), door-to-needle (DTN), and door-to-groin (DTG) times. On a comparison with the standard data of NoCOVID times, we found that OTD was significantly longer (*F*: 6.94; *p* value: 0.001) (Table 5). No difference was found in OTD, OTN, and OTG between COVID time—1 and COVID time—2 patients.

**Table 5:** Time interval of emergent reperfusion therapies. *OTD*, onset-to-door; *DTN*, door-to-needle (IVT); *DTG*, door-to-groin (MT)

Reperfusion therapies	OTD (min)	DTN (min)	DTG (min)
(*n*)	mean ± SD		
COVID time (51)	145.1 ± 79.4 *	105.7 ± 33.7	146.2 ± 58.2
NoCOVID time—1 (48)	112.8 ± 107.7	102.1 ± 39.3	147.2 ± 87.4
NoCOVID time—2 (47)	83.7 ± 45.2	109.7 ± 28.5	142.1 ± 34.5

*p value: 0.001, OTD in the COVID vs. NoCOVID times

### Increase in cerebral hemorrhages

To investigate this phenomenon, we analyzed a series of clinical variables (arterial hypertension, antiplatelet and anticoagulant therapy, location, size) potentially conditioning or characterizing the occurrence of cerebral hemorrhages. However, we found no significant result (Table 6).

**Table 6:** Clinical variables associated with intracerebral hemorrhages. *AntiPLT*, antiplatelet; *NOAs*, new oral anticoagulants; *ED*, emergency department

Total hemorrhages	Arterial hypertension	Known hypertension in ED	Unknown hypertension in ED	AntiPLT agents	NOAs	“Typical” location	Diameter > 4 cm
(*n*)	(*n*, % hemorrhages)						
**COVID** time (56)	38; 67.9%	20; 35.7%	6; 10.7%	21; 37.5%	6; 10.7%	24; 42.9%	20; 35.7%
**NoCOVID** time—1 (33)	26; 78.8%	11; 33.3%	2; 6.1%	18; 54.5%	1; 3%	14; 42.4%	15; 45.5%
**NoCOVID** time—2 (41)	32; 78.1%	14; 34.1%	2; 4.8%	17; 41.5%	6; 14.6%	18; 43.9%	9; 22%

## Discussion

We described the experience of our tertiary referral Neurology Unit in the management of neurological emergencies during the COVID-19 pandemic, in which context we considered a time span (8 months) longer than many earlier studies [2–4]. However, our definition of the COVID time onset and offset suffered from some approximation due to the monthly counts and observation restraints.

As reported by other Italian and international studies [3, 5–7], our Institution’s overall accesses to the ED were cut down by about 40% during the COVID time. Many intuitive factors are advocated to explain the phenomenon, such as the public appeal not to crowd the EDs during the lockdown or the natural fear of infection in the hospital environment [8].

The percentage of hospital admissions following an ED access grew dramatically due to COVID cases, while admissions to the Neurology Unit were slightly but non-significantly reduced (6.3% compared with 7.3% of the NoCOVID times). This finding implicitly reflects the severe nature of urgent neurological presentations, often requiring in-hospital care at all times.

This is even more evident for ischemic stroke, which is the most common reason for admission to the Neurology Unit. Many studies from different countries reported a significant drop in the ischemic stroke incidence, though many were limited to the “first” wave of the COVID-19 pandemic [4, 9–12]. The present local series based on a longer observation time does not reach the same conclusions, supporting the view that ischemic stroke has remained a major issue in the emergency setting, even during COVID times.

Human and material resources from many departments, including those of Neurology, have been reallocated worldwide to face the COVID-19 pandemic, limiting operative capacities [13]. As a result, at our Institution, the Stroke Unit lost its usual physical location and most of its dedicated personnel. Nevertheless, such occurrence had just a marginal, non-significant effect on the patient inflow and reperfusion treatments. The new locations for stroke patients were suboptimal, but possibly they did not compromise the clinical practice substantially. Intravenous thrombolysis (IVT), mechanical thrombectomy (MT), and bridge therapies were carried out nearly as usual, through extraordinary efforts and unavoidable difficulties on the medical and paramedical side. These results are partially at variance with those already published [4, 14]. The Italian Stroke Organization performed a multicenter survey involving 93 Italian Stroke Units—including data from March 2020 (“first COVID-19 wave”)—reporting a generalized sharp reduction in hospitalizations for cerebrovascular events as well as a decrease in the number of IVT, while endovascular MT remained unchanged or increased [4]. These findings are in line with those from other groups, reporting a reduction in the IVT proportion compared to a stable proportion of MT in the first 3 months of the COVID-19 pandemic [14]. On the contrary, other authors described a reduction of IVT and MT in COVID time following the general reduction in ischemic stroke admissions [11]. In our opinion, the decrease in IVT is most likely a consequence of delayed presentation at the emergency department at the beginning of the pandemic, thus exceeding the narrow time window for treatment. On the contrary, the time window for mechanical thrombectomy is broader, allowing more patients to receive treatment. This view is in line with our findings, which show that the OTD interval was significantly (*p* value: 0.001) longer during the COVID time. Other authors reported similar data [15].

Apparently, the stroke care at the “Maggiore della Carità” Hospital, Novara, Italy, did not suffer significant damage from the COVID time, as far as the number of patients and their treatment are concerned. This conclusion may depend on the sample size we considered, which did not allow us to show statistically significant differences. Another important factor may well be the longer observation time in comparison with previous studies [4, 14]. Time may have allowed the intra-hospital stroke network to better adapt to limitations and changes imposed by the pandemic. This is confirmed by our findings of unchanged door-to-needle and door-to-groin times on the overall COVID time. On the other hand, a separate analysis of COVID time—1, when adaptation to the pandemic hardships was lacking, did not reveal a significant disruption of the in-hospital stroke approach. These results may just reflect local, small-scale phenomena, which are not in contradiction, and may coexist, with the threat facing stroke care in the COVID era.

Besides emergent reperfusion treatments, stroke care is a network linking prevention strategies, territory emergency systems, and in-hospital care in Stroke Units with well-established procedures and qualified stroke specialists, as well as rehabilitation/long-term care facilities. Therefore, it is likely that despite the best efforts, the COVID-19 pandemic will have, to some extent, affected the long-term outcome of stroke patients.

At variance with previous work [4, 16], we recorded an unexpected increase in primary intracerebral hemorrhage (*p* value: 0.02) during the COVID time. We attempted to identify any difference from NoCOVID times in pathogenic or characteristic clinical variables, but we found none. It has been reported that the COVID-19 pandemic represented a powerful stressor in the general population, while access to primary care decreased, affecting the monitoring of chronic conditions such as hypertension [17]. We speculate this might underlie unrecognized hypertensive episodes.

During the COVID time, no change was detected in terms of hospitalization for epileptic diseases, including epileptic status, in accordance with previous reports [18]. The same was true for cerebral neoplasms. Nevertheless, these pathologies obviously represent another compelling urgency for the patient to access the hospital structures.

By contrast, presentations such as “vertigo” were virtually abolished, and this may reflect the over/misuse of this diagnosis to cover milder conditions of dizziness and unsteadiness, which may not require invariable access to the ED.

## Conclusion

Through an extended analysis of the COVID times (8 months), we found that the overall urgent admissions to our Neurology Unit showed just a mild and non-significant reduction. The same was true for the number of ischemic strokes admitted. The number of reperfusion procedures remained substantially unchanged. Yet, the onset-to-door time was significantly longer (*p* value: 0.001). Unexpectedly, a significant (*p* value: 0.02) increase in intracerebral hemorrhages emerged, a phenomenon that lacks immediate explanations. A regular Stroke Unit was unavailable, which might affect the prognosis of stroke in the long term.

## Data Availability

The datasets generated during the analysis of this study are available from the corresponding author on reasonable request.
